# Poor sleep quality and later sleep timing are risk factors for osteopenia and sarcopenia in middle-aged men and women: The NEO study

**DOI:** 10.1371/journal.pone.0176685

**Published:** 2017-05-01

**Authors:** Eliane A. Lucassen, Renée de Mutsert, Saskia le Cessie, Natasha M. Appelman-Dijkstra, Frits R. Rosendaal, Diana van Heemst, Martin den Heijer, Nienke R. Biermasz, for the NEO study group

**Affiliations:** 1 Laboratory for Neurophysiology, Department of Molecular Cell Biology, Leiden, Leiden University Medical Center, The Netherlands; 2 Department of Internal Medicine, The Hague, Medisch Centrum Haaglanden, The Netherlands; 3 Department of Clinical Epidemiology, Leiden, Leiden University Medical Center, The Netherlands; 4 Department of Medical Statistics, Leiden, Leiden University Medical Center, The Netherlands; 5 Department of Endocrinology, Leiden, Leiden University Medical Center, The Netherlands; 6 Department of Gerontology and Geriatrics, Leiden, Leiden University Medical Center, The Netherlands; 7 Department of Clinical Epidemiology, Leiden, Leiden University Medical Centre, The Netherlands; 8 Department of Internal Medicine, Amsterdam, VU Medical Centre, The Netherlands; Nanjing Medical University, CHINA

## Abstract

**Context:**

Sleep deprivation has detrimental metabolic consequences. Osteopenia and sarcopenia usually occur together and increase risk of fractures and disease. Results from studies linking sleep parameters to osteopenia or sarcopenia are scarce and inconsistent.

**Objective:**

To examine the associations of sleep parameters with osteopenia and sarcopenia, considering the influence of sex and menopause.

**Design, setting and participants:**

Cross-sectional analysis of 915 participants (45–65 years, 56% women, BMI 26 (range: 18–56) kg/m^2^) in the Netherlands Epidemiology of Obesity (NEO) study, a population-based cohort study. Sleep duration, quality, and timing were assessed with the Pittsburgh Sleep Quality Index (PSQI); bone mineral density and relative appendicular muscle mass were measured by DXA scans. Linear and logistic regressions were performed to associate sleep parameters to bone mineral density, relative appendicular muscle mass, osteopenia (t-score between -1 and -2.5) and sarcopenia (1 SD below average muscle mass).

**Results:**

After adjustment for confounding factors, one unit increase in PSQI score (OR and 95% CI, 1.09, 1.03–1.14), declined self-rated sleep quality (1.76, 1.03–3.01), sleep latency (1.18, 1.06–1.31), and a one hour later sleep timing (1.51, 1.08–2.11), but not sleep duration (1.05, 0.90–1.23), were associated with osteopenia. PSQI score (1.10, 1.02–1.19) was also associated with sarcopenia; OR’s of sleep latency and later mid-sleep time with sarcopenia were 1.14 (0.99–1.31) and 1.54 (0.91–2.61), respectively. Associations were somewhat stronger in women and varied per menopausal status.

**Conclusions:**

These results suggest that decreased sleep quality and a later sleep timing are risk factors for osteopenia and sarcopenia in middle aged individuals.

## Introduction

More than one in three individuals report unintentionally falling asleep during the daytime, implying that insufficient sleep is a prevalent problem [1]. Sleep deprivation has profound effects on health, including increased cardiovascular disease, diabetes, and obesity, which may be caused by alterations in sympatho-vagal balance, increased evening cortisol levels, pro-inflammatory changes, and/or decreased levels of growth hormone [2]. These effects are also observed in studies that selectively reduced sleep quality without affecting total sleep duration [3,4]. Additionally, sleep timing may impact health, as evening types generally have higher levels of catecholamines and cortisol [5,6].

These endocrine, neural and inflammatory alterations mentioned above have the potential to negatively affect the musculoskeletal system. Recent studies suggest that insufficient sleep is associated with a decline in musculoskeletal parameters, although outcomes have been inconsistent [7,8]. Studies have reported positive [9,10], negative [11,12] or U-shaped [13] associations between sleep duration and bone mineral density (BMD), or observed no association [7]. One small study observed a negative association between sleep duration and skeletal muscle mass [8]. Self-reported sleep quality has been associated with an increased risk of osteoporosis [14] and fractures [15,16], but in these studies sleep quality was assessed by one single question. Other studies observed that individuals with a later sleep timing were at increased risk of osteoporosis (BMD ≥2.5 SD below young reference population) [10,17] and sarcopenia (low muscle mass) [18].

Osteopenia (BMD 1–2.5 SD below young reference population) and sarcopenia (relative skeletal muscle mass >1 SD below young reference population) are prevalent conditions, with 22% and 36% of middle-aged men and women classifying as osteopenic [19] and 27% and 35% of middle-aged men and women as sarcopenic [20]. These frequently concurrent conditions become more prevalent with age and enhance each other’s negative effects on health [21]. Fracture risk increases more dramatically with age than would be suspected by the corresponding decline in BMD alone [22], likely due to the simultaneous age-related decline in muscle mass [23–25]. At present there has been no study on the association between sleep parameters, bone mass and muscle mass simultaneously, despite the fact that these parameters are related and important factors for musculoskeletal health [23–25]. Therefore, we performed a study addressing the associations between sleep duration, sleep timing and sleep quality and osteopenia and sarcopenia in a population based cohort study. Furthermore we examined to what extent these associations differ between men and women and by menopausal status.

## Materials and methods

### Study design and study population

The Netherlands Epidemiology of Obesity (NEO) study is a population-based prospective cohort study that included 6,671 participants between September 2008 and September 2012. This study was designed to investigate pathways leading to disease in overweight and obese individuals. Men and women aged between 45 and 65 years with a self-reported BMI of 27 kg/m^2^ or higher from the city of Leiden and its surrounding municipalities (the Netherlands) were eligible to participate and were recruited via registries, general practitioners, and advertisements. In addition, all inhabitants aged between 45 and 65 years from one municipality (Leiderdorp) were invited irrespective of their BMI, allowing for a reference distribution of BMI. Participants were invited for a baseline visit at the NEO study center of the Leiden University Medical Center (LUMC) after an overnight fast.

The present study is a cross-sectional analysis of data obtained at the baseline visit. Prior to this visit, all participants completed questionnaires with demographic, lifestyle, and clinical information, in addition to a questionnaire on sleep behaviour. At the baseline visit, anthropometric measurements and fasting blood samples were obtained in the morning after participants fasted for at least 10 hours. Furthermore, a random subsample of 915 participants (56% women) underwent dual energy X-ray absorptiometry (DXA) measurements, assessing bone mineral densitometry (BMD), body fat mass and lean body mass. For the present analysis, we only included participants with DXA measurements. Individuals with bedtimes between 05:00 and 20:00 hrs were excluded from sleep timing analyses (n = 4). The study design and population have been described more extensively previously [26].

This study was approved by the medical ethics committee of the Leiden University Medical Center (LUMC) and all participants provided written informed consent.

### Data collection

Ethnicity, education, menopausal status, medication use, and past fractures were self-reported on the questionnaire. Education was considered high if participants highest level of education was higher vocational school, university, or post-graduate education. Menopausal status was divided into three categories (pre-, peri-, and postmenopausal) based on information on oopho- or hysterectomy and/or self-reported menopausal status. Women who reported a hysterectomy were classified according to their age (premenopausal when <46 years, perimenopausal when 46 to 55 years, and postmenopausal when ≥55 years). Women who did not report menopausal status and were 58 years or older were classified as postmenopausal. In addition, all use of medication in the month preceding the study visit was recorded.

BMI was calculated from the height, measured with a fixed and calibrated tape measure, and the weight, measured without shoes and one kilogram was subtracted to correct for the weight of clothing. Waist and hip circumference were measured mid-way between the lower costal margin and the iliac crest and at the maximal circumference of the buttocks, respectively.

Habitual alcohol intake in grams per day was assessed using a semi-quantitative food frequency questionnaire (FFQ), which has been validated in the Dutch population [27], and calculated from the 2011 version of the Dutch food composition table [28].

Participants reported the frequency and duration of their usual physical activity in the past 4 weeks on the Short Questionnaire to Assess Health-enhancing physical activity, a method previously validated in the Dutch population [29,30]. We calculated the energy expended during physical activity in leisure time in hours per week of metabolic equivalents.

Serum vitamin D 25(OH) concentrations were measured with LC-MSMS calibrated 2nd generation electrochemoluminescence immunoassay [26].

### Sleep measurements

The Pittsburg Sleep Quality Index (PSQI) was completed at home prior to the baseline visit [31]. It includes questions about the average estimated duration of actual sleep and the usual bedtime and risetime during the past month. We defined the mid-sleep time as the clock time when half of the total sleep duration was completed, calculated from the usual bedtime and risetime. The questionnaire also contained questions concerning self-rated sleep quality (“very good”, “fairly good”, “fairly bad”, or “very bad”), sleep latency (range: 0–6 units) and sleep duration. The total PSQI score ranges from 0 to 21, with higher scores indicating a worse sleep quality.

### DXA bone and muscle measurements

Absolute BMD values were obtained for the lumbar spine (L1-L4) and the proximal femur regions (Hologic Discovery A, Tromp Medical BV, Castricum, the Netherlands). Mean vertebral (∑ L1-4 BMD / 4) and mean hip BMD (left BMD + right BMD / 2) were calculated for each individual. When the left or right hip measurement was missing (n = 10; 1.1%), we used the available side for analysis. T-scores were calculated based on the number of standard deviations below the BMD of a younger reference population of the NHANES III taking sex and ethnicity into account [32]. A t-score between -1 and -2.5 in either hip or vertebrae was considered as osteopenic, and -2.5 or lower as osteoporotic [17]. Whole body fat mass (kg) was assessed from the total body DXA scans.

The relative appendicular skeletal muscle mass (RASM) was calculated by summing the lean mass of the arms and legs and was corrected for body weight by division by the whole body mass as measured by DXA. Sarcopenia was defined as a RASM of one standard deviation below the RASM of a young reference population (RASM below 29.9% and 25.1% for men and women, respectively) [33].

### Statistical analysis

Stata version 12.1 was used for analyses (StataCorp LP, TX, US). In the NEO study, individuals with a BMI of 27 kg/m^2^ or higher were oversampled. To correctly represent associations in the general population [34], adjustments for the oversampling of individuals with a BMI ≥ 27 kg/m^2^ were made. This was done by weighting all participants towards the BMI distribution of participants from the Leiderdorp municipality [35], whose BMI distribution was similar to the BMI distribution of the general Dutch population [36]. All results were based on weighted analyses. Consequently, the results apply to a population-based study without oversampling of participants with a BMI ≥ 27 kg/m^2^.

Baseline characteristics of the weighted study population are given in mean (SD), median (interquartile range) for skewed variables, or as proportion of the study population. Pearson correlations between muscle and bone parameters were performed. Associations between categorical variables were assessed with Chi square tests. We performed linear regression analyses to examine the associations between sleep parameters and bone and muscle mass, expressed in mean differences with 95% confidence intervals (CI). In addition, we performed logistic regression analyses to estimate odds ratios and 95% CI for osteopenia and sarcopenia associated with the sleep parameters. Crude models, models adjusted for age, sex and total body fat, and models additionally adjusted for ethnicity, education, alcohol intake, physical activity, concentrations of vitamin D, season of the year, use of systemic corticosteroids and bisphosphonates, and for menopausal status in women were constructed. Finally, to examine whether sleep parameters were associated with osteopenia and sarcopenia conditional on muscle mass and BMD, respectively, we additionally adjusted the models of osteopenia for muscle mass and models of sarcopenia for BMD. We examined whether associations differed in men and women and by menopausal status in women by testing interaction terms with sex and also stratified all analyses by sex and menopausal status.

## Results

Of 6,671 middle-aged men and women of the NEO cohort, 915 participants who underwent DXA were included in present analysis. Included participants were 59 SD 4 years of age (range: 45–65 years), had a mean BMI of 25.9 (IQR: 23.0–27.9) kg/m^2^, and 56% were women. Participants who underwent DXA scans averagely had a 9% higher BMI and had a 8% lower PSQI score than other NEO participants.

Baseline characteristics of participants are presented in **Table 1**, stratified by sex. Men smoked more often, had a higher BMI, but had less body fat (as measured by DXA) than women. BMD in the spine and the hip and RASM were highest in men. 38% and 5% had osteopenia or osteoporosis in the hip or spine, respectively. The presence of osteopenia and osteoporosis did not differ between men and women, while the prevalence of sarcopenia was lowest in men. Men had less sleep latency, but lower sleep quality measures and later sleep times than women.

**Table 1:** Baseline characteristics of participants of the Netherlands Epidemiology of Obesity study, aged 45 to 65 years and stratified by sex.

	Men	Women
***General characteristics***		
Age in years	56 (6)	55 (6)
Ethnicity in % Caucasian	95	93
Current smoker in %	16	10
Former smoker in %	44	43
Education in % high^1^	50	46
BMI kg/m^2^	27 (4)	25 (5)
Whole body fat mass in kg	24 (7)	27 (9)
Whole body fat in % of total body weight	26 (5)	36 (6)
Premenopausal in %	N.A.	20
Perimenopausal in %	N.A.	22
Postmenopausal in %	N.A.	58
Age of menopause in years	N.A.	50 (4)
Habitual alcohol consumption in g/day	14 (6–26)	7 (1–14)
Physical activity in MET-h/week	26 (14–46)	27 (17–49)
History of fractures in %	41	31
Vitamin D 25(OH) in nmol/l	58 (25)	63 (28)
Usage vitamin D supplements in %	4	7
Usage bisphosphonates in %	0	1
Usage calcium supplements in %	2	10
Usage systemic corticoids in %	1	1
Usage any sleeping medication in %	4	5
Usage benzodiazepines in %	2	4
Usage cyclopyrrolones in %	1	1
Usage melatonin in %	0.4	1
Usage homeopathic sleeping medication in %	0.2	0
***Sleep characteristics***		
PSQI total score; range: 0–21	4.1 (2.6)	5.1 (3.2)
Self-rated sleep quality “very good” in %	32	21
Self-rated sleep quality “fairly good” in %	55	62
Self-rated sleep quality “fairly bad” in %	13	15
Self-rated sleep quality “very bad” in %	1	1
Sleep latency; range: 0–6	0 (0–1)	1 (0–3)
Sleep latency in min	10 (5–15)	15 (10–30)
Usual bedtime in hh:mm and min	23:22 (53)	23:10 (46)
Usual mid-sleep time in hh:mm and min	3:50 (30)	4:05 (31)
Usual rise time in hh:mm and min	7:03 (66)	7:21 (56)
Sleep duration in hrs	7.0 (1.0)	7.1 (1.0)
***Bone and muscle characteristics***		
BMD spine in g/cm^2^	1.1 (0.1)	1.0 (0.1)
BMD hip in g/cm^2^	1.0 (0.1)	0.9 (0.1)
BMD spine t-score	-0.4 (1.3)	-0.5 (1.3)
BMD hip t-score	-0.1 (0.8)	-0.3 (1.0)
Osteopenic in spine in %	30	29
Osteopenic in hip in %	13	24
Osteopenic in hip or spine in %	35	40
Osteoporotic in spine in %	4	6
Osteoporotic in hip in %	0	0
Osteoporotic in hip or spine in %	4	6
ASM in kg	30 (4)	19 (3)
RASM in % of body mass	33 (3)	27 (3)
Sarcopenic in %	9	27

^1^high education: higher vocational school, university, or post-graduate education. BMI, body mass index; MET, metabolic equivalents; PSQI, Pittsburgh Sleep Quality Index; C1, Component 1; C2, Component 2, BMD, bone mineral density; ASM, appendicular skeletal muscle mass; RASM, relative appendicular skeletal muscle mass. Data are presented as mean (SD), median (25th-75th percentile) or percentage. Results were based on analyses weighted towards the BMI distribution of the general population (n = 512 women, n = 403 men).

Mean difference in percent RASM of total body weight per gram/cm^2^ BMD was 3.37 (CI 95%: 1.25–5.47) and in the spine 8.93 (6.87–10.99) in the hip. The prevalence of sarcopenia was 29% for participants with a normal BMD, 29% for those with osteopenia and 29% for those with osteoporosis. Of the participants with sarcopenia, 32% had osteopenia and 4% had osteoporosis. However, osteopenia was not associated with sarcopenia (OR 0.94, CI 95% 0.66–1.32).

### Associations of sleep parameters with BMD and muscle mass

**Table 2** shows the mean differences in BMD in the spine and hip and muscle mass (RASM) associated with one unit change in the sleep parameters. After adjustment for potential confounding factors, measures of sleep quality were weakly associated with BMD in the spine and hip. For example, per unit in PSQI score (range: 0–21, higher values represent worsened sleep quality) BMD was 0.003 g/m^2^ reduced in the spine and hip, respectively (about 0.3% / PSQI unit of BMD of the average participant). With each unit increase in sleep latency (range: 0–6 units, increasing values represent increasing sleep latency) BMD in the spine was 0.007 g/m^2^ lower (0.7% / sleep latency unit of BMD of the average participant).

**Table 2:** Difference (95% CI) in BMD spine, BMD hip and RASM per unit change of sleep parameter in the whole cohort.

*Sleep measure*	BMD spine in g/cm^2^	BMD hip in g/cm^2^	RASM in % of total body weight
***Crude models***			
PSQI score	-0.005	-0.006	-0.057
	(-0.008 - -0.001)	(-0.009 - -0.003)	(-0.088 - -0.027)
Self-rated sleep quality			
Very good (reference)			
Fairly good	-0.025	-0.039	-0.922
	(-0.049 - -0.001)	(-0.059 –-0.019)	(-1.620 - -0.225)
Fairly or	-0.029	-0.023	-1.202
very bad	(-0.063 - -0.004)	(-0.055–0.010)	(-2.180 - -0.225)
Sleep latency score	-0.012	-0.013	-0.627
	(-0.019 - -0.006)	(-0.019 - -0.013)	(-0.810 - -0.444)
Mid-sleep time in hrs	-0.036	-0.049	-0.644
	(-0.057 - -0.015)	(-0.067 - -0.007)	(-0.822 - -0.465)
Sleep duration in hrs	-0.011 (-0.021–0.000)	-0.010 (-0.020 - -0.001)	-0.125 (-0.214 - -0.035)
***Age-*, *sex-*, *and whole body fat mass-adjusted models***			
PSQI score	-0.004	-0.003	-0.011
	(-0.007–0.000)	(-0.006–0.000)	(-0.029–0.007)
Self-rated sleep quality			
Very good (reference)	-	-	-
Fairly good	-0.011	-0.018	-0.112
	(-0.034–0.012)	(-0.036–0.001)	(-0.397–0.173)
Fairly or	-0.022	-0.022	-0.081
very bad	(-0.053–0.009)	(-0.034–0.021)	(-0.479–0.317)
Sleep latency score	-0.007	-0.002	-0.070
	(-0.013 - -0.001)	(-0.008–0.003)	(-0.155–0.014)
Mid-sleep time in hrs	-0.014	-0.012	-0.139
	(-0.035–0.006)	(-0.028–0.004)	(-0.246 - -0.032)
Sleep duration in hrs	-0.006 (-0.016–0.004)	-0.004 (-0.012–0.005)	-0.031 (-0.080–0.019)
***Maximally adjusted models***			
PSQI score	-0.003	-0.003	-0.026
	(-0.007–0.000)	(-0.006–0.000)	(-0.076–0.023)
Self-rated sleep quality			
Very good (reference)	-	-	-
Fairly good	-0.017	0.012	-0.165
	(-0.035–0.001)	(-0.035–0.011)	(-0.442–0.112)
Fairly or	-0.006	-0.020	-0.002
very bad	(-0.034–0.023)	(-0.051–0.010)	(-0.396–0.392)
Sleep latency score	-0.007	-0.003	-0.061
	(-0.014 - -0.001)	(-0.008–0.002)	(-0.146–0.025)
Mid-sleep time in hrs	-0.014	-0.010	-0.091
	(-0.035–0.006)	(-0.026–0.007)	(-0.344–0.162)
Sleep duration in hrs	-0.008	-0.004	-0.027
	(-0.018–0.002)	(-0.013–0.005)	(-0.102–0.156)

Crude; age, sex, whole body fat mass adjusted; and maximally adjusted (age, sex, whole body fat mass ethnicity, education, alcohol intake, physical activity, vitamin D levels and season, usage of systemic corticosteroids and bisphosphonates) models are shown. PSQI, Pittsburgh Sleep Quality Index; BMD, bone mineral density; RASM, relative appendicular skeletal muscle mass. Results were based on analyses weighted towards the BMI distribution of the general population (n = 512 women, n = 403 men).

In analyses stratified by sex, increased PSQI score was inversely associated with BMD in women, and not in men (**Table A in S1 File**). Likewise, poor self-rated sleep quality associated with reduced BMD in women only. Sleep duration was associated with lumbar spine BMD only in men: a one hour decline in sleep duration related to a 2% reduced BMD.

Additional analyses were stratified by menopausal status (**Table B in S1 File**). Of all women, 20% were premenopausal, 22% were perimenopausal and 58% were postmenopausal. The associations between PSQI score and change in BMD were somewhat stronger in pre- and perimenopausal (between 0.7% and 1.0% of BMD of average participants) women than in postmenopausal women.

### Sleep parameters and risk of osteopenia and sarcopenia

**Fig 1:**
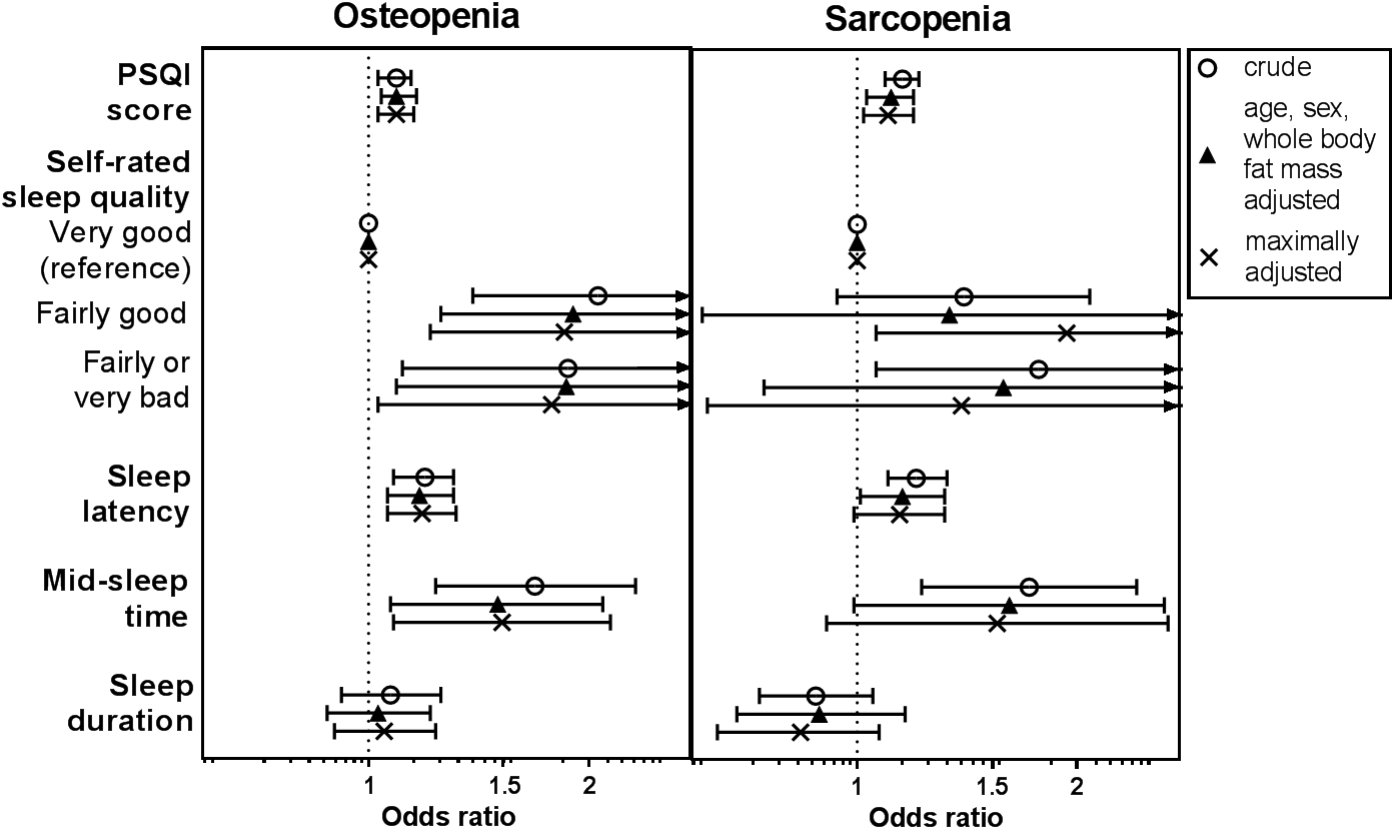
OR (95% CI) for osteopenia and sarcopenia per unit change of sleep parameter. Crude; age, sex, whole body fat mass adjusted; and maximally adjusted (age, sex, whole body fat mass, ethnicity, education, alcohol intake, physical activity, vitamin D levels and season, and usage of systemic corticosteroids and bisphosphonates) models are shown. PSQI, Pittsburgh Sleep Quality Index. Results were based on analyses weighted towards the BMI distribution of the general population (n = 915).

Odds ratios for the association of sleep parameters with osteopenia and sarcopenia are shown in **Fig 1**. In our middle-aged study population, only 4% of men and 6% of women were classified as osteoporotic, which precluded reliable estimation of the relationship between sleep parameters and osteoporosis. Of all participants, 38% had osteopenia in either the hip or the spine (35% of men and 40% of women) and 19% participants had sarcopenia (9% of men and 27% of women). The prevalence of osteopenia and sarcopenia differed per menopausal status. Of pre-, peri- and postmenopausal women, 26%, 28% and 50% had osteopenia, and 18%, 24%, and 31% had sarcopenia, respectively.

In the models adjusted for putative confounding factors, sleep quality and sleep timing were associated with higher odds of osteopenia and sarcopenia. Each unit increase in PSQI score (indicating worse sleep quality) was associated with 9% and 10% increased risk of osteopenia and sarcopenia, respectively (OR 1.09, 1.03–1.15; OR 1.10, 1.02–1.19). A “fairly bad” or “very bad” self-rated sleep quality associated with a 76% increased risk of osteopenia compared to a “very good” self-rated sleep quality (OR 1.76, 1.03–3.01). A unit increase in sleep latency score (indicating a longer sleep latency; range: 0–6) was associated with both osteopenia (OR 1.18, 1.06–1.31) and sarcopenia (OR 1.14, 0.99–1.31). Mid-sleep time was associated with osteopenia (OR 1.51, 1.08–2.11), while the odds radio for sarcopenia was 1.54 (0.91–2.61). The OR’s of sleep duration with osteopenia and sarcopenia in the whole study population were 1.05 (0.90–1.23), and 0.84 (0.65–1.07), respectively.

When individuals with osteoporosis were included in the analyses results were similar (**Table C in S1 File**). Additionally, sleep parameters associated similarly with osteopenia in the spine and in the hip, although the mid-sleep time associated somewhat stronger with osteopenia in the hip than with osteopenia in the spine (**Table C in S1 File**).

### Odds ratios of osteopenia and sarcopenia stratified by sex and menopausal status

**Fig 2:**
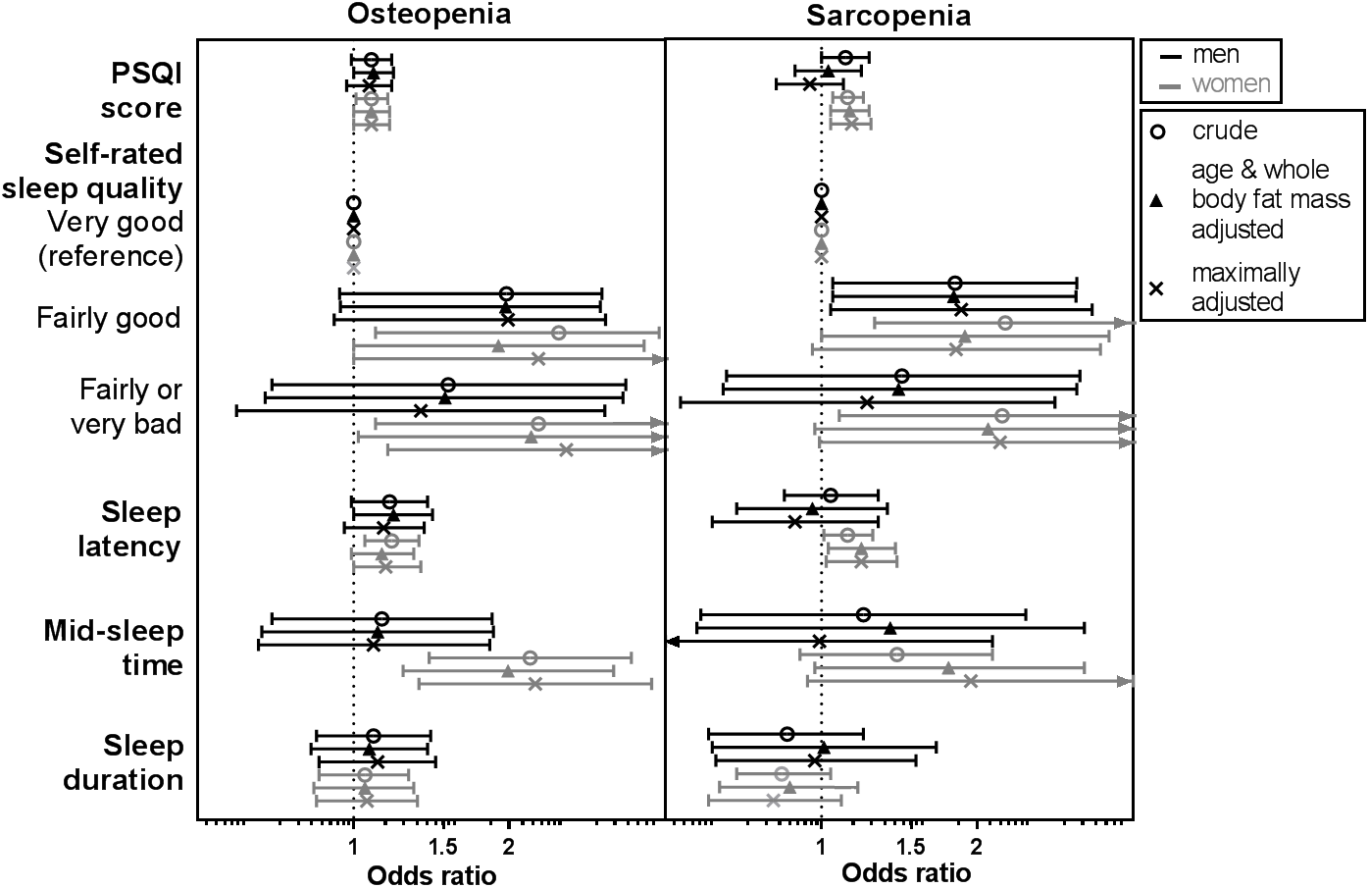
OR (95% CI) for osteopenia and sarcopenia per unit change of sleep parameter stratified by sex. Crude, age & whole body fat mass adjusted, and maximally adjusted (age, whole body fat mass ethnicity, education, alcohol intake, physical activity, vitamin D levels and season, usage of systemic corticosteroids and bisphosphonates, and menopause) models are shown. PSQI, Pittsburgh Sleep Quality Index. Results were based on analyses weighted towards the BMI distribution of the general population (n = 915).

In sex-stratified analyses most associations were stronger in women (**Fig 2**). For PSQI and sleep latency, associations between men and women were only marginally different for osteopenia. For example, a unit increase in PSQI score associated with a 7% and 8% higher risk for osteopenia in men (OR 1.07, 0.97–1.18) and in women (OR 1.08, 1.00–1.17). Correspondingly, a one-unit increased sleep latency score (range 0–6) associated with a 14% and 15% higher risk for osteopenia in men (OR 1.14, 0.96–1.36) and women (OR 1.15, 1.00–1.34). For sarcopenia however, one unit higher PSQI score associated with a 14% increased risk in women (OR 1.14, 1.04–1.24) a longer sleep latency with a 19% higher risk (OR 1.19, 1.02–1.39), but these sleep parameters were not associated with sarcopenia in men (PSQI score OR 0.95, 0.82–1.10; sleep latency OR 0.89, 0.62–1.28). For self-rated sleep quality and mid-sleep time, associations were stronger in women than in men for osteopenia and for sarcopenia. Similarly, women rating their sleep quality as “fairly or very bad” instead of “very good” had over a two-fold risk of osteopenia (OR 2.53, 1.16–5.51) and sarcopenia (OR 2.18, 0.99–4.82), while this was not associated with musculoskeletal parameters in men (osteopenia OR 1.34, 0.60–2.99; sarcopenia OR 1.22, 0.54–2.77). Sleep duration did not affect the risk of osteopenia or sarcopenia.

In analysis stratified by menopausal status (**Fig A in S1 File**), the association between mid-sleep time and osteopenia appeared to be strongest in premenopausal women, while PSQI score and sleep latency in postmenopausal women were associated mainly with sarcopenia. Associations between sleep duration and osteopenia and sarcopenia were not observed in any of the menopausal groups.

### Sleep parameters and risk of osteopenia adjusted for muscle mass and of sarcopenia adjusted for BMD

After additional adjustment of the models for osteopenia and sarcopenia for muscle mass and BMD, respectively, associations did not change in more than trivial ways (**Fig B in S1 File**).

## Discussion

In this population-based study, we extensively examined associations of sleep duration, sleep timing and sleep quality with osteopenia and sarcopenia in middle-aged men and women. In our cohort, a decline in sleep quality measures was associated with an increased risk of osteopenia and sarcopenia. All measures of sleep quality (total PSQI score, self-reported sleep quality, and sleep latency) were consistently associated with musculoskeletal health.

Individuals with a later sleep timing also have an increased risk of osteopenia and sarcopenia. The mid-sleep time is a marker for chronotype, which constitutes the individual timing of behavior caused by the internal circadian phase relative to the light-dark cycle. The evening chronotype has been associated with higher levels of cortisol and of catecholamines [2,6], which could in turn negatively affect the musculoskeletal system. These high levels of cortisol and catecholamines may be caused by the discrepancy between preferred and actual sleep timing that evening types often experience due to social constraints [37]. Chronic circadian misalignment has been related to metabolic derangements previously [38,39]. For instance, circadian disruption induced by constant light worsened bone microstructure in young mice resembling age-related osteoporosis [40]. Alternatively, effects of sleep time on the musculoskeletal system may be partly explained by behavioral differences. Individuals exhibiting later sleep times display more unhealthy behaviors, such as smoking, increased alcohol usage [41], less physical activity [42], and later meal times [5]. However, associations between mid-sleep time and osteopenia and sarcopenia remained present after adjusting analyses for smoking and physical activity. Our results are consistent with studies that showed that postmenopausal women with bedtimes after midnight were at increased risk for osteoporosis [10] and that sarcopenia was most prevalent in middle-aged individuals with a later chronotype [18].

The association between sleep quality and osteopenia is in accordance with existing literature, that has reported associations between sleep quality and osteoporosis [14] and fractures [15, 16]. Interestingly, the associations with sleep quality and timing with musculoskeletal health were stronger in women than in men, especially for mid-sleep time. Sex differences exist in the prevalence of osteopenia and sarcopenia [19,20] and in sleep parameters, with middle aged women tending to sleep longer (17 minutes in [43]) and spending more time in slow wave sleep than men [43,44]. The relationship between sleep and musculoskeletal health may also be influenced by sex-related differences in sex hormones, employment status, and social behavior. We observed some minor differences per menopausal status. The associations between sleep quality and timing and osteopenia were somewhat weaker in postmenopausal than in premenopausal women, possibly because the lack of estrogen after the menopause exerts effects bone loss to such a strong degree [45] that bone loss becomes less dependent on sleep. The association between the PSQI score and sarcopenia was strongest in postmenopausal women, so sleep quality does associate with muscle mass while these women are losing muscle rapidly due to the loss of estrogen [46]. However, groups were small and these results need to be confirmed in larger cohorts.

We did not observe an association between sleep duration and osteopenia or sarcopenia. Based on experimental sleep deprivation studies, we expected that short sleep would elicit changes in mediators that could negatively affect musculoskeletal health, such as decreased IGF-1 levels and increased stress hormones [2]. However, few individuals (9%) slept less than the recommended 6 hrs per night [47] and more severe sleep deprivation may be necessary to induce such effects [2]. Furthermore, our results are consistent with a study in 20 to 66 year old Americans that also did not observe an association between sleep duration and BMD [7]. Other studies that have shown inconsistent results were mostly performed in Asians [9–13]. It is possible that previous results were inconsistent because there is an optimum in sleep duration (the association is U shaped) and associations are age-dependent. It has also been reported that long sleeping postmenopausal women have a reduced skeletal muscle mass [8], while we did not observe this in postmenopausal women. In that study women slept on average 8.7 hrs while in ours this was 7.0 hrs, which may account for the differences.

Associations between sleep quality and bone quality were similar in the spine and hip. Trabecular bone (typical in the spine) has a more rapid rate of age- and postmenopausal-related deterioration than cortical bone (typical in the hip) and is more responsive to cortisol [48], suggesting that the associations between sleep and bone may be independent of these factors.

In line with previous studies [22,23,49] BMD was associated with muscle mass in our study. However, we did not observe an association between osteopenia and sarcopenia. This is somewhat surprising, since there is evidence that deterioration of the musculoskeletal system is characterized by bone loss and muscle wasting in parallel and that these processes share common determinants [21, 50], but may be explained by the relatively young age of our participants and the therefore low prevalence of osteopenia/sarcopenia. Therefore, the mechanisms linking sleep quality/timing with osteopenia and sarcopenia may be different. Exhibiting both conditions will greatly increase the risk for fractures and frailty [22,23].

There are several pathophysiological mechanisms that may explain the relation between sleep and musculoskeletal health. One mechanism is *via* endocrine factors known to be affected by sleep and influencing musculoskeletal health. A decline in sleep quality may prevent individuals from reaching the deepest sleep stages [3], which are thought to be the most “restorative” and these are the times at which most GH is secreted [51]. GH exerts effects on the musculoskeletal system directly and through mediating insulin-like growth factor I (IGF-1) levels [52]. GH and IGF-1 levels progressively decline in aging and are important contributors to age-associated osteopenia and sarcopenia [52,53]. In addition to GH, studies that experimentally fragmented sleep reported higher cortisol levels and a higher sympathovagal balance [2–4], which can exert detrimental effects on the musculoskeletal system directly and by inhibiting GH and IGF-1 [5,52]. Additionally, observational studies report a positive association IGF-1 levels and amount spent in deep sleep [54,55].

We observed that sleep quality measures associated only weakly with BMD in the spine and hip, implying that there may be a cut-off point of more progressed stages of bone and muscle loss that are associated with insufficient sleep.

A limitation of this study is that it is cross-sectional, so we cannot exclude residual confounding and reverse causation. Osteopenia and sarcopenia may have led to reduced sleep quality. Furthermore, likely due to their young age [56], only 5% of participants in our cohort had osteoporosis, which prevented us to reliably determine the associations of sleep parameters with osteoporosis. Nevertheless, the majority of individuals with osteopenia will likely develop osteoporosis in the next 10 years [57]. The method of calculating RASM by dividing the lean mass of the extremities by the whole body mass provides some interpretation difficulties: in a person with a relatively higher fat mass, RASM may be overestimated, so relative fat mass may have influenced results. However, an increase in body weight is also reflected in the lean mass and RASM is the most commonly used method for assessing muscle mass because the absolute lean mass is less informative [20,33]. Although a widely accepted definition for sarcopenia is still lacking, the geriatric syndrome of sarcopenia often includes functional assessments of muscle strength, which our study did not include. Although we assessed sleep quality with an extensive questionnaire, whereas previous studies linked sleep to bone health with one single question [14–16], the use of sleep diaries and actigraphy may be even more reliable methods to assess sleep. Furthermore, the use of chronotype questionnaires may more reliably assess preference for sleep timing than mid-sleep time alone [58].

Strengths of the study were the reliable assessment of BMD and muscle mass by DXA, in contrast to quantitative ultrasound [13,14] or bioimpedance analysis [8], in a large study population, and extensive measurement of potential confounding factors at baseline, including demographic factors, body composition, alcohol intake, physical activity, vitamin D levels and medication usage. Furthermore, the inclusion of assessing both osteopenia and sarcopenia allowed us to determine their relative associations with sleep parameters and to identify potential interactions.

Taken together, decreased sleep quality and a later sleep timing contribute to increased risk of osteopenia and sarcopenia in the middle-aged. Future studies should examine the associations of sleep with musculoskeletal health and fracture risk. It has been demonstrated that sleep quality can be improved by the implementation of personalized sleep hygiene strategies [59], offering a pathway for sleep interventional studies to study the causative effect of sleep on musculoskeletal health.

## Supporting information

S1 File **Table A in S1 File: Difference (95% CI) in BMD spine, BMD hip and RASM per unit change of sleep parameter stratified by sex.** Crude, age & whole body fat mass adjusted, and maximally adjusted (age, whole body fat mass ethnicity, education, alcohol intake, physical activity, vitamin D levels and season, usage of systemic corticosteroids and bisphosphonates, and menopause) models are shown. ^1^age, whole body fat mass, ethnicity, education, alcohol intake, physical activity, menopause. PSQI, Pittsburgh Sleep Quality Index; BMD, bone mineral density; RASM, relative appendicular skeletal muscle mass. Results were based on analyses weighted towards the BMI distribution of the general population (n = 915). **Table B in S1 File: Difference (95% CI) in BMD spine, BMD hip and RASM per unit change of sleep parameter stratified by menopause.** Crude, age & whole body fat mass adjusted, and maximally adjusted (age, whole body fat mass, ethnicity, education, alcohol intake, physical activity, vitamin D levels and season, usage of systemic corticosteroids and bisphosphonates) models are shown. PSQI, Pittsburgh Sleep Quality Index; BMD, bone mineral density; RASM, relative appendicular skeletal muscle mass. Results were based on analyses weighted towards the BMI distribution of the general population (n = 915). **Table C in S1 File: OR (95% CI) for osteopenia in spine or hip, osteopenia and osteoporosis in spine or hip, osteopenia in the spine, and osteopenia in the hip per unit change of sleep parameter.** Crude; age, sex, and whole body fat mass adjusted; and maximally adjusted (age, whole body fat mass, ethnicity, education, alcohol intake, physical activity, vitamin D levels and season, usage of systemic corticosteroids and bisphosphonates) models are shown. PSQI, Pittsburgh Sleep Quality Index. Results were based on analyses weighted towards the BMI distribution of the general population (n = 915). **Fig A in S1 File: Difference (95% CI) in BMD spine, BMD hip and RASM per unit change of sleep parameter stratified by menopausal status.** OR (95% CI) for osteopenia and sarcopenia per unit change of sleep parameter stratified per menopausal status. Crude; age & whole body fat mass adjusted; and maximally adjusted (age, whole body fat mass, ethnicity, education, alcohol intake, physical activity, vitamin D levels and season, usage of systemic corticosteroids and bisphosphonates) models are shown. PSQI, Pittsburgh Sleep Quality Index. Results were based on analyses weighted towards the BMI distribution of the general population (n = 915). **Fig B in S1 File: OR (95% CI) for osteopenia and sarcopenia per unit change of sleep parameter additionally adjusted for muscle mass and BMD, respectively.** OR (95% CI) for osteopenia and sarcopenia per unit change of sleep parameter. Maximally adjusted models are shown for reference (age, sex, whole body fat mass, ethnicity, education, alcohol intake, physical activity, vitamin D levels and season, usage of systemic corticosteroids and bisphosphonates (note that these models are also shown in **Fig 1**)), and models were additionally adjusted for muscle mass, BMD spine, or BMD hip. PSQI, Pittsburgh Sleep Quality Index; BMD, bone mineral density. Results were based on analyses weighted towards the BMI distribution of the general population (n = 915).
(DOC)
