# Supplementary material for: Poor sleep quality and later sleep timing are risk factors for osteopenia and sarcopenia in middle-aged men and women: The NEO study
Source: PLoS One. 2017 May 1;12(5):e0176685. doi: 10.1371/journal.pone.0176685 (PMC5411054; doi:10.1371/journal.pone.0176685)
Supplement: S1 File — Table A in S1 File: Difference (95% CI) in BMD spine, BMD hip and RASM per unit change of sleep parameter stratified by sex. Crude, age & whole body fat mass adjusted, and maximally adjusted (age, whole body fat mass ethnicity, education, alcohol intake, physical activity, vitamin D levels and season, usage of systemic corticosteroids and bisphosphonates, and menopause) models are shown. 1age, whole body fat mass, ethnicity, education, alcohol intake, physical activity, menopause. PSQI, Pittsburgh Sleep Quality Index; BMD, bone mineral density; RASM, relative appendicular skeletal muscle mass. Results were based on analyses weighted towards the BMI distribution of the general population (n = 915). Table B in S1 File: Difference (95% CI) in BMD spine, BMD hip and RASM per unit change of sleep parameter stratified by menopause. Crude, age & whole body fat mass adjusted, and maximally adjusted (age, whole body fat mass, ethnicity, education, alcohol intake, physical activity, vitamin D levels and season, usage of systemic corticosteroids and bisphosphonates) models are shown. PSQI, Pittsburgh Sleep Quality Index; BMD, bone mineral density; RASM, relative appendicular skeletal muscle mass. Results were based on analyses weighted towards the BMI distribution of the general population (n = 915). Table C in S1 File: OR (95% CI) for osteopenia in spine or hip, osteopenia and osteoporosis in spine or hip, osteopenia in the spine, and osteopenia in the hip per unit change of sleep parameter. Crude; age, sex, and whole body fat mass adjusted; and maximally adjusted (age, whole body fat mass, ethnicity, education, alcohol intake, physical activity, vitamin D levels and season, usage of systemic corticosteroids and bisphosphonates) models are shown. PSQI, Pittsburgh Sleep Quality Index. Results were based on analyses weighted towards the BMI distribution of the general population (n = 915). Fig A in S1 File: Difference (95% CI) in BMD spine, BMD hip and RASM per unit change of s [file pone.0176685.s001.doc]

# S1 File

# Table A

Difference (95% CI) in BMD spine, BMD hip and RASM per unit change of sleep parameter stratified by sex.

| ***Sleep measure*** | **BMD spine in g/cm^2^** | | **BMD hip in g/cm^2^** | | **RASM in % of total body weight** | |
| --- | --- | --- | --- | --- | --- | --- |
|  | **men** | **women** | **men** | **women** | **men** | **women** |
| ***Crude models*** | | | | | | |
| PSQI score | 0.000 | -0.005 | 0.001 | -0.005 | -0.089 | -0.106 |
|  | (-0.005 - 0.006) | (-0.010 - -0.001) | (-0.004 - 0.006) | (-0.008 - -0.001) | (-0.187 - 0.009) | (-0.218 - 0.005) |
| Self-rated sleep quality score |  |  |  |  |  |  |
|  |  |  |  |  |  |  |
| Very good |  |  |  |  |  |  |
| (reference) | - | - | - | - | - | - |
| Fairly good | 0.004 | -0.039 | 0.004 | -0.054 | -0.056 | -0.050 |
|  | (-0.029 – 0.036) | (-0.073 - -0.005) | (-0.029 -0.036) | (-0.081 - -0.027) | (-0.623 – 0.510) | (-0.734 – 0.635) |
| Fairly or | 0.003 | -0.045 | 0.022 | -0.034 | -0.140 | -0.362 |
| very bad | (-0.045 – 0.051) | (0.089 - -0.011) | (-0.023 – 0.066) | (-0.075 – 0.007) | (-0.978 – 0.697) | (-1.302 – 0.579) |
| Sleep latency score | -0.002 | -0.011 | 0.004 | -0.008 | -0.154 | -0.073 |
|  | (-0.013 - 0.009) | (-0.019 - -0.003) | (-0.006 - 0.015) | (-0.015 - -0.002) | (-0.344 - 0.037) | (-0.257 - 0.112) |
| Mid-sleep time in hrs | -0.005 | -0.037 | -0.014 | -0.033 | 0.018 | 0.022 |
|  | (-0.038 - 0.027) | (-0.066 - -0.009) | (-0.039 - 0.011) | (-0.057 - -0.009) | (-0.010 - 0.047) | (-0.004 - 0.048) |
| Sleep duration in hrs | -0.014  (-0.030 - 0.001) | -0.005  (-0.019 - 0.009) | -0.011  (-0.024 - 0.002) | -0.005  (-0.017 - 0.008) | 0.073  (-0.202 - 0.349) | 0.190  (-0.091 - 0.472) |
|  | | | | | | |
| ***Age & whole body fat mass-adjusted models*** | | | | | | |
| PSQI score | -0.001 | -0.005 | 0.000 | -0.004 | -0.035 | -0.039 |
|  | (-0.006 - 0.005) | (-0.009 - 0.000) | (-0.005 - 0.004) | (-0.008 - 0.000) | (-0.088 - 0.019) | (-0.108 - 0.030) |
| Self-rated sleep quality score |  |  |  |  |  |  |
|  |  |  |  |  |  |  |
| Very good |  |  |  |  |  |  |
| (reference) | - | - | - | - | - | - |
| Fairly good | 0.003 | -0.022 | 0.004 | -0.038 | -0.001 | -0.202 |
|  | (-0.028 – 0.035) | (-0.054 – 0.011) | (-0.022 -0.030) | (-0.063 - -0.013) | (-0.366 – 0.364) | (-0.640 – 0.235) |
| Fairly or | 0.004 | -0.038 | 0.017 | -0.028 | -0.232 | -0.042 |
| very bad | (-0.043 – 0.050) | (-0.081 – 0.004) | (-0.024 – 0.058) | (-0.066 – 0.010) | (-0.724 – 0.259) | (-0.638 – 0.554) |
| Sleep latency score | -0.003 | -0.006 | 0.003 | -0.004 | -0.111 | -0.055 |
|  | (-0.014 - 0.008) | (-0.014 - 0.002) | (-0.008 - 0.013) | (-0.010 - 0.002) | (-0.234 - 0.012) | (-0.167 - 0.056) |
| Mid-sleep time in hrs | -0.004 | -0.023 | -0.003 | -0.019 | -0.035 | -0.101 |
|  | (-0.037 - 0.028) | (-0.050 - 0.004) | (-0.028 - 0.022) | (-0.040 - 0.002) | (-0.454 - 0.383) | (-0.457 - 0.256) |
|  |  |  |  |  |  |  |
|  |  |  |  |  |  |  |
| Sleep duration in hrs | -0.013  (-0.028 - 0.002) | -0.003  (-0.016 - 0.010) | -0.006  (-0.019 - 0.007) | -0.003  (-0.015 - 0.008) | -0.002  (-0.189 - 0.185) | 0.044  (-0.147 - 0.235) |
|  | | | | | | |
| ***Maximally adjusted models^1^*** | | | | | | |
| PSQI score | 0.001 | -0.004 | 0.001 | -0.006 | -0.007 | -0.043 |
|  | (-0.005 - 0.007) | (-0.009 - -0.000) | (-0.004 - 0.005) | (-0.008 - -0.001) | (-0.064 - 0.050) | (-0.114 - 0.028) |
| Self-rated sleep quality score |  |  |  |  |  |  |
|  |  |  |  |  |  |  |
| Very good |  |  |  |  |  |  |
| (reference) | - | - | - | - | - | - |
| Fairly good | 0.000 | -0.023 | 0.002 | -0.040 | -0.015 | -0.379 |
|  | (-0.031 – 0.032) | (-0.055 – 0.009) | (-0.024 – 0.028) | (-0.064 – 0.159) | (-0.371 – 0.341) | (-0.800 – 0.041) |
| Fairly or | 0.001 | -0.042 | 0.025 | -0.035 | -0.020 | 0.073 |
| very bad | (-0.043 – 0.057) | -(0.082 – -0.002) | (-0.020 – 0.071) | (-0.071 – 0.002) | (-0.479 – 0.439) | (-0.679 – 0.532) |
| Sleep latency score | -0.002 | -0.007 | 0.003 | -0.005 | -0.054 | -0.063 |
|  | (-0.013 - 0.009) | (-0.015 - 0.001) | (-0.008 - 0.013) | (-0.011 - 0.002) | (-0.176 - 0.067) | (-0.178 - 0.051) |
| Mid-sleep time in hrs | -0.004 | -0.025 | -0.001 | -0.019 | 0.035 | -0.181 |
|  | (-0.036 - 0.027) | (-0.052 - 0.002) | (-0.025 - 0.028) | (-0.040 - 0.002) | (-0.332 - 0.402) | (-0.505 - 0.144) |
| Sleep duration in hrs | -0.017 | -0.005 | -0.008 | -0.003 | -0.003 | 0.055 |
|  | (-0.032 - -0.001) | (-0.017 - 0.008) | (-0.022 - 0.005) | (-0.014 - 0.008) | (-0.174 - 0.168) | (-0.127 - 0.238) |

Crude, age & whole body fat mass adjusted, and maximally adjusted (age, whole body fat mass ethnicity, education, alcohol intake, physical activity, vitamin D levels and season, usage of systemic corticosteroids and bisphosphonates, and menopause) models are shown. ^1^age, whole body fat mass, ethnicity, education, alcohol intake, physical activity, menopause. PSQI, Pittsburgh Sleep Quality Index; BMD, bone mineral density; RASM, relative appendicular skeletal muscle mass. Results were based on analyses weighted towards the BMI distribution of the general population (n = 512 women, n = 403 men).

**Table B:**

Difference (95% CI) in BMD spine, BMD hip and RASM per unit change of sleep parameter stratified by menopausal status.

| ***Sleep measures*** | **BMD spine in g/cm^2^** | | | | **BMD hip in g/cm^2^** | | | **RASM in % of total body weight** | | | |
| --- | --- | --- | --- | --- | --- | --- | --- | --- | --- | --- | --- |
|  | **premenopausal** | **perimenopausal** | | **postmenopausal** | **premenopausal** | **perimenopausal** | **postmenopausal** | **premenopausal** | **perimenopausal** | **postmenopausal** | |
| ***Crude models*** | | | | | | | | | | | |
| PSQI score | -0.004 | | -0.004 | -0.003 | -0.004 | -0.007 | -0.001 | -0.117 | -0.231 | -0.028 | |
|  | (-0.018 - 0.010) | | (-0.011 - 0.004) | (-0.009 - 0.003) | (-0.016 - 0.008) | (-0.014 - -0.001) | (-0.006 - 0.003) | (-0.421 - 0.187) | (-0.379 - -0.083) | (-0.178 - 0.122) | |
| Self-rated sleep quality score |  | |  |  |  |  |  |  |  |  | |
|  |  |  |  |  |  |  |  |  |  |  |  |
| Very good |  | |  |  |  |  |  |  |  |  | |
| (reference) | - | | - | - | - | - | - | - | - | - | |
| Fairly good | -0.011 | | -0.018 | 0.035 | -0.037 | -0.040 | -0.047 | 0.458 | -0.323 | 0.144 | |
|  | (-0.072 – 0.051) | | (-0.067 – 0.031) | (-0.086 – 0.016) | (-0.094 – 0.020) | (-0.088 – 0.008) | (-0.081 - -0.013) | (-1.022 – 1.939) | (-1.79 – 1.14) | (-0.770 – 1.057) | |
| Fairly or | -0.046 | | -0.019 | -0.038 | 0.003 | -0.036 | -0.023 | 0.493 | -0.417 | -0.214 | |
| very bad | (-0.163 – 0.071) | | (0.102 – 0.065) | (-0.096 – 0.020) | (-0.066 – 0.071) | (-0.128 – 0.057) | (-0.070 – 0.025) | (-2.547 – 3.533) | (-2.153 - 1.320) | (-1.455 – 1.027) | |
| Sleep latency score | -0.012 | | -0.004 | -0.006 | -0.004 | -0.007 | -0.003 | -0.197 | -0.132 | 0.048 | |
|  | (-0.040 - 0.016) | | (-0.018 - 0.010) | (-0.016 - 0.004) | (-0.029 - 0.022) | (-0.022 - 0.009) | (-0.011 - 0.004) | (-0.775 - 0.381) | (-0.439 - 0.176) | (-0.185 - 0.282) | |
| Mid-sleep time hrs | -0.045 | | -0.021 | -0.026 | -0.047 | -0.017 | -0.022 | 0.887 | 0.021 | 0.021 | |
|  | (-0.101 - 0.011) | | (-0.078 - 0.036) | (-0.065 - 0.012) | (-0.103 - 0.009) | (-0.068 - 0.034) | (-0.051 - 0.008) | (-0.936 - 2.710) | (-0.843 - 0.884) | (-0.843 - 0.884) | |
| Sleep duration in hrs | -0.018 | | -0.012 | -0.001 | -0.013 | -0.006 | -0.005 | 0.056 | 0.447 | 0.062 | |
|  | (-0.048 - 0.012) | | (-0.033 - 0.008) | (-0.019 - 0.018) | (-0.043 - 0.017) | (-0.031 - 0.019) | (-0.019 - 0.009) | (-0.657 - 0.769) | (0.059 - 0.834) | (-0.358 - 0.482) | |
|  | | | | | | | | | | | |
| ***Age- and whole body fat mass-adjusted models*** | | | | | | | | | | | |
| PSQI score | -0.006 | | -0.006 | -0.003 | -0.007 | -0.01 | -0.001 | 0.011 | -0.136 | | -0.018 |
|  | (-0.019 - 0.006) | | (-0.014 - 0.001) | (-0.009 - 0.003) | (-0.017 - 0.003) | (-0.016 - -0.004) | (-0.006 - 0.004) | (-0.170 - 0.193) | (-0.217 - -0.056) | | (-0.113 - 0.077) |
| Self-rated sleep quality score |  | |  |  |  |  |  |  |  | |  |
|  |  |  |  |  |  |  |  |  |  |  |  |
| Very good |  | |  |  |  |  |  |  |  | |  |
| (reference) | - | | - | - | - | - | - | - | - | | - |
| Fairly good | -0.006 | | -0.003 | 0.029 | -0.032 | -0.025 | -0.042 | 0.145 | -0.915 | | -1.660 |
|  | (-0.064 – 0.051) | | (-0.049 – 0.044) | (-0.081 – 0.024) | (-0.082 – 0.018) | (-0.065 – 0.016) | (-0.077 - -0.008) | (-0.826 – 1.120) | (-1.737 - -0.093) | | (-0.699 – 0.367) |
| Fairly or | -0.040 | | -0.016 | -0.043 | 0.010 | -0.035 | -0.029 | 0.337 | -0.491 | | -0.089 |
| very bad | (-0.153 – 0.073) | | (-0.095 – 0.063) | (-0.101 – 0.016) | (-0.061 – 0.080) | (-0.118 – 0.049) | (-0.076 – 0.017) | (1.605 – 2.279) | (-1.457 – 0.474) | | (-0.862 – 0.684) |
| Sleep latency score | -0.012 | | -0.004 | -0.004 | -0.003 | -0.007 | -0.001 | -0.102 | -0.119 | | -0.028 |
|  | (-0.038 - 0.014) | | (-0.018 - 0.010) | (-0.014 - 0.006) | (-0.026 - 0.021) | (-0.021 - 0.006) | (-0.008 - 0.006) | (-0.453 - 0.250) | (-0.306 - 0.067) | | (-0.168 - 0.112) |
| Mid-sleep time in hrs | -0.038 | | -0.019 | -0.022 | -0.039 | -0.016 | -0.015 | 0.353 | -0.037 | | -0.037 |
|  | (-0.088 - 0.011) | | (-0.071 - 0.032) | (-0.061 - 0.017) | (-0.083 - 0.005) | (-0.061 - 0.029) | (-0.042 - 0.013) | (-0.797 - 1.503) | (-0.620 - 0.546) | | (-0.620 - 0.546) |
| Sleep duration in hrs | -0.014 | | -0.006 | 0.001 | -0.009 | 0.001 | -0.004 | -0.158 | 0.221 | | 0.017 |
|  | (-0.041 - 0.012) | | (-0.027 - 0.015) | (-0.018 - 0.019) | (-0.036 - 0.018) | (-0.024 - 0.025) | (-0.018 - 0.010) | (-0.651 - 0.336) | (-0.026 - 0.468) | | (-0.257 - 0.292) |
|  | | | | | | | | | | | |
| ***Maximally adjusted models*** | | | | | | | | | | | |
| PSQI score | -0.009 | -0.010 | | 0.000 | -0.011 | -0.007 | -0.001 | -0.111 | -0.132 | | -0.002 |
|  | (-0.019 - 0.001) | (-0.015 - -0.004) | | (-0.005 - 0.005) | (-0.023 - 0.002) | (-0.015 - -0.000) | (-0.007 - 0.005) | (-0.292 - 0.070) | (-0.224 - -0.039) | | (-0.101 - 0.098) |
| Self-rated sleep quality score |  | |  |  |  |  |  |  |  | |  |
|  |  |  |  |  |  |  |  |  |  |  |  |
| Very good |  | |  |  |  |  |  |  |  | |  |
| (reference) | - | | - | - | - | - | - | - | - | | - |
| Fairly good | -0.015 | | -0.006 | -0.016 | -0.035 | -0.025 | -0.034 | -0.330 | -0.803 | | -0.142 |
|  | (-0.075 – 0.044) | | (-0.050 – 0.038) | (-0.066 – 0.034) | (-0.083 – 0.014) | (-0.067 – 0.017) | (-0.068 - -0.000) | (-1.180 – 0.519) | (-1.667 – 0.062) | | (-0.701 – 0.418) |
| Fairly or | -0.068 | | 0.051 | -0.022 | -0.023 | -0.058 | -0.019 | -0.239 | -0.502 | | 0.193 |
| very bad | (-0.174 – 0.039) | | (-0.114 – 0.013) | (-0.079 – 0.034) | (-0.080 – 0.034) | (-0.131 – 0.016) | (-0.065 – 0.026) | (-2.165 – 1.572) | (-1.545 – 0.541) | | (-0.650 – 1.036) |
| Sleep latency score | -0.002 | -0.010 | | 0.000 | -0.013 | -0.007 | -0.002 | -0.141 | -0.130 | | -0.012 |
|  | (-0.023 - 0.019) | (-0.023 - 0.003) | | (-0.008 - 0.007) | (-0.038 - 0.012) | (-0.020 - 0.006) | (-0.012 - 0.008) | (-0.467 - 0.186) | (-0.330 - 0.069) | | (-0.163 - 0.138) |
| Mid-sleep time in hrs | -0.035 | -0.021 | | -0.019 | -0.029 | -0.036 | -0.027 | -0.232 | -0.054 | | -0.362 |
|  | (-0.075 - 0.006) | (-0.069 - 0.027) | | (-0.046 - 0.007) | (-0.089 - -0.032) | (-0.089 - 0.018) | (-0.064 - 0.011) | (-0.559 – 1.024) | (-0.769 - 0.662) | | (-0.826 - 0.101) |
| Sleep duration in hrs | 0.009 | -0.001 | | -0.008 | 0.007 | 0.008 | -0.005 | 0.173 | 0.236 | | -0.047 |
|  | (-0.012 - 0.030) | (-0.024 - 0.026) | | (-0.022 - 0.005) | (-0.026 - 0.040) | (-0.029 - 0.013) | (-0.023 - 0.012) | (-0.305 - 0.652) | (-0.058 - 0.531) | | (-0.328 - 0.233) |

Crude, age & whole body fat mass adjusted, and maximally adjusted (age, whole body fat mass, ethnicity, education, alcohol intake, physical activity, vitamin D levels and season, usage of systemic corticosteroids and bisphosphonates) models are shown. PSQI, Pittsburgh Sleep Quality Index; BMD, bone mineral density; RASM, relative appendicular skeletal muscle mass. Results were based on analyses weighted towards the BMI distribution of the general population (n = 512 women, n = 403 men).

# Table C:

OR (95% CI) for osteopenia in the spine or hip (note: same values as depicted in **Fig. 1**), osteopenia or osteoporosis in the spine or hip, osteopenia in the spine, and osteopenia in the neck per unit change of sleep parameter.

| ***Sleep measures*** | **Osteopenia spine or hip** | **Osteopenia or osteoporosis spine or hip** | **Osteopenia in spine** | **Osteopenia in hip** |
| --- | --- | --- | --- | --- |
| ***Crude*** | | | | |
| PSQI score | 1.09 | 1.08 | 1.06 | 1.09 |
|  | (1.03 - 1.14) | (1.03 - 1.14) | (1.01 - 1.12) | (1.03 - 1.16) |
| Self-reported sleep quality |  |  |  |  |
| Very good   (reference) | - | - | - | - |
| Fairly good | 2.03 | 1.95 | 1.78 | 2.38 |
|  | (1.38-2.99) | (1.34 - 2.85) | (1.18 - 2.69) | (1.39 - 4.10) |
| Fairly or | 1.85 | 1.73 | 1.86 | 1.69 |
| very bad | (1.11-3.08) | (1.04 - 2.87) | (1.08 - 3.20) | (0.82 – 3.50) |
| Sleep latency | 1.19 | 1.18 | 1.12 | 1.22 |
| score | (1.08 - 1.30) | (1.07 - 1.29) | (1.01 - 1.23) | (1.10 - 1.36) |
| Mid sleep | 1.67 | 1.62 | 1.16 | 2.01 |
| time in hrs | (1.23 - 2.28) | (1.19 - 2.19) | (0.86 - 1.58) | (1.35 - 2.97) |
| Sleep | 1.07 | 1.07 | 1.00 | 1.11 |
| duration in hrs | (0.92 - 1.25) | (0.92 - 1.24) | (0.85 - 1.18) | (0.91 - 1.36) |
|  | | | | |
| ***Age, sex, whole body fat mass corrected*** | | | | |
| PSQI score | 1.09 | 1.09 | 1.07 | 1.09 |
|  | (1.04 - 1.16) | (1.04 - 1.16) | (1.02 - 1.14) | (1.01 - 1.17) |
| Self-reported sleep quality |  |  |  |  |
| Very good   (reference) | - | - | - | - |
| Fairly good | 1.98 | 1.92 | 1.75 | 2.36 |
|  | (1.33 - 2.95) | (1.30 - 2.83) | (1.16 - 2.66) | (1.35 – 4.14) |
| Fairly or | 1.93 | 1.82 | 1.92 | 1.80 |
| very bad | (1.15 - 3.24) | (1.09 – 3.04) | (1.11 - 3.32) | (0.86 - 3.78) |
| Sleep latency | 1.17 | 1.17 | 1.13 | 1.15 |
| score | (1.06 - 1.30) | (1.06 - 1.30) | (1.02 - 1.26) | (1.01 - 1.31) |
| Mid sleep | 1.49 | 1.44 | 1.12 | 1.62 |
| time in hrs | (1.07 - 2.06) | (1.04 – 2.00) | (0.81 - 1.56) | (1.04 - 2.54) |
| Sleep | 1.03 | 1.03 | 0.98 | 1.05 |
| duration in hrs | (0.88 - 1.21) | (0.88 - 1.20) | (0.83 - 1.16) | (0.84 - 1.32) |
|  | | | | |
| ***Maximally adjusted*** | | | | |
| PSQI score | 1.09 | 1.08 | 1.07 | 1.10 |
|  | (1.03 - 1.15) | (1.02 - 1.15) | (1.01 - 1.13) | (1.01 - 1.19) |
| Self-reported sleep quality |  |  |  |  |
| Very good   (reference) | - | - | - | - |
| Fairly good | 1.97 | 1.90 | 1.75 | 2.42 |
|  | (1.31 - 2.97) | (1.27 - 2.84) | (1.14 - 2.68) | (1.36 - 4.30) |
| Fairly or | 1.97 | 1.76 | 1.88 | 1.99 |
| very bad | (1.10 - 3.20) | (1.03 – 2.98) | (1.07 - 3.31) | (0.93 – 4.26) |
| Sleep latency | 1.18 | 1.18 | 1.13 | 1.17 |
| score | (1.06 - 1.31) | (1.06 - 1.31) | (1.02 - 1.25) | (1.02 - 1.33) |
| Mid sleep | 1.51 | 1.47 | 1.11 | 1.58 |
| time in hrs | (1.08 - 2.11) | (1.05 - 2.05) | (0.80 - 1.54) | (1.00 - 2.51) |
| Sleep | 1.05 | 1.05 | 0.99 | 1.02 |
| duration in hrs | (0.90 - 1.23) | (0.90 - 1.23) | (0.85 - 1.17) | (0.81 - 1.28) |

Crude; age, sex, and whole body fat mass adjusted; and maximally adjusted (age, whole body fat mass, ethnicity, education, alcohol intake, physical activity, vitamin D levels and season, usage of systemic corticosteroids and bisphosphonates) models are shown. PSQI, Pittsburgh Sleep Quality Index. Results were based on analyses weighted towards the BMI distribution of the general population (n = 512 women, n = 403 men).

#
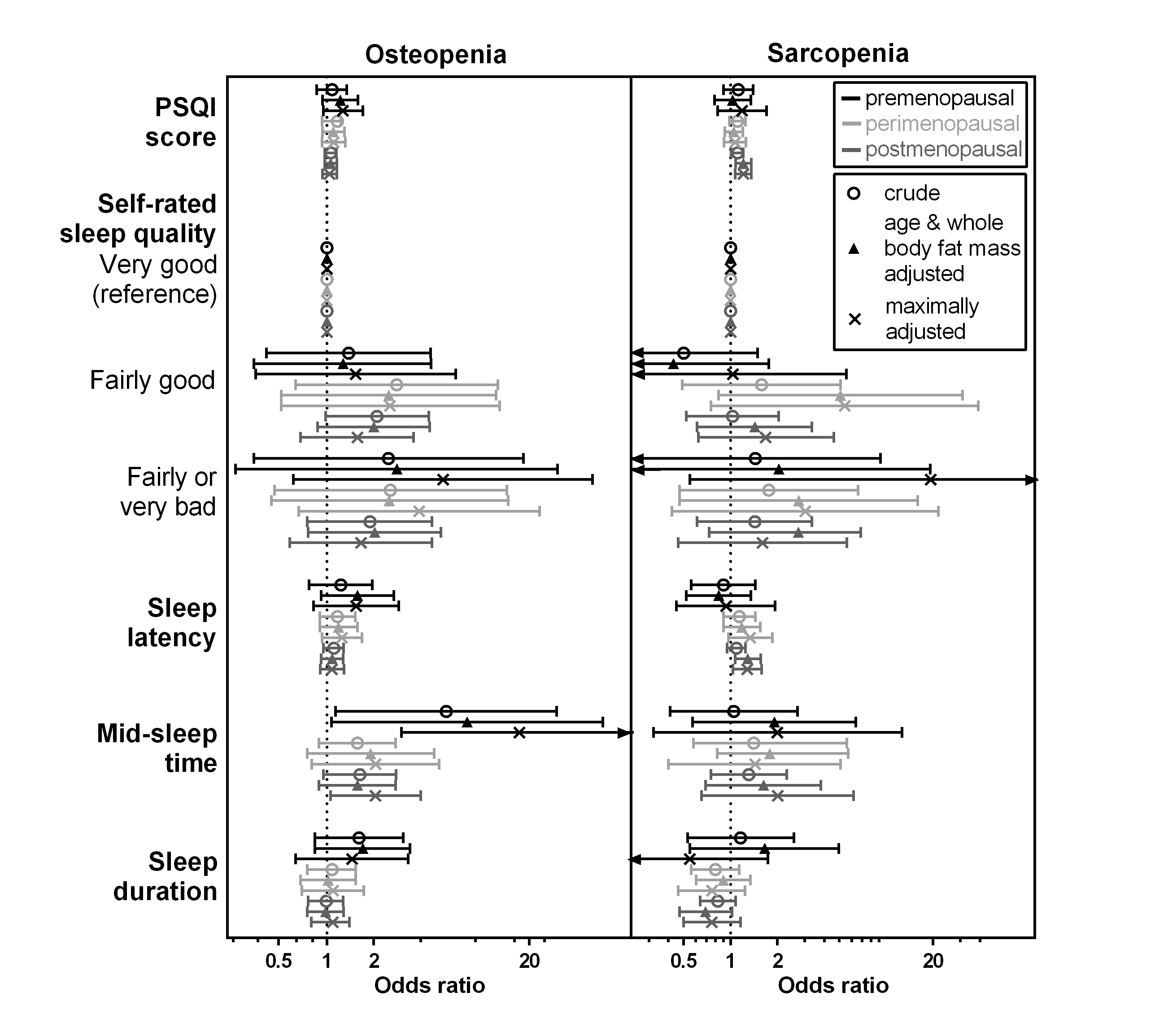
 Fig A:

OR (95% CI) for osteopenia and sarcopenia per unit change of sleep parameter stratified per menopausal status. Crude; age & whole body fat mass adjusted; and maximally adjusted (age, whole body fat mass, ethnicity, education, alcohol intake, physical activity, vitamin D levels and season, usage of systemic corticosteroids and bisphosphonates) models are shown. PSQI, Pittsburgh Sleep Quality Index. Results were based on analyses weighted towards the BMI distribution of the general population (n = 512 women, n = 403 men).

#
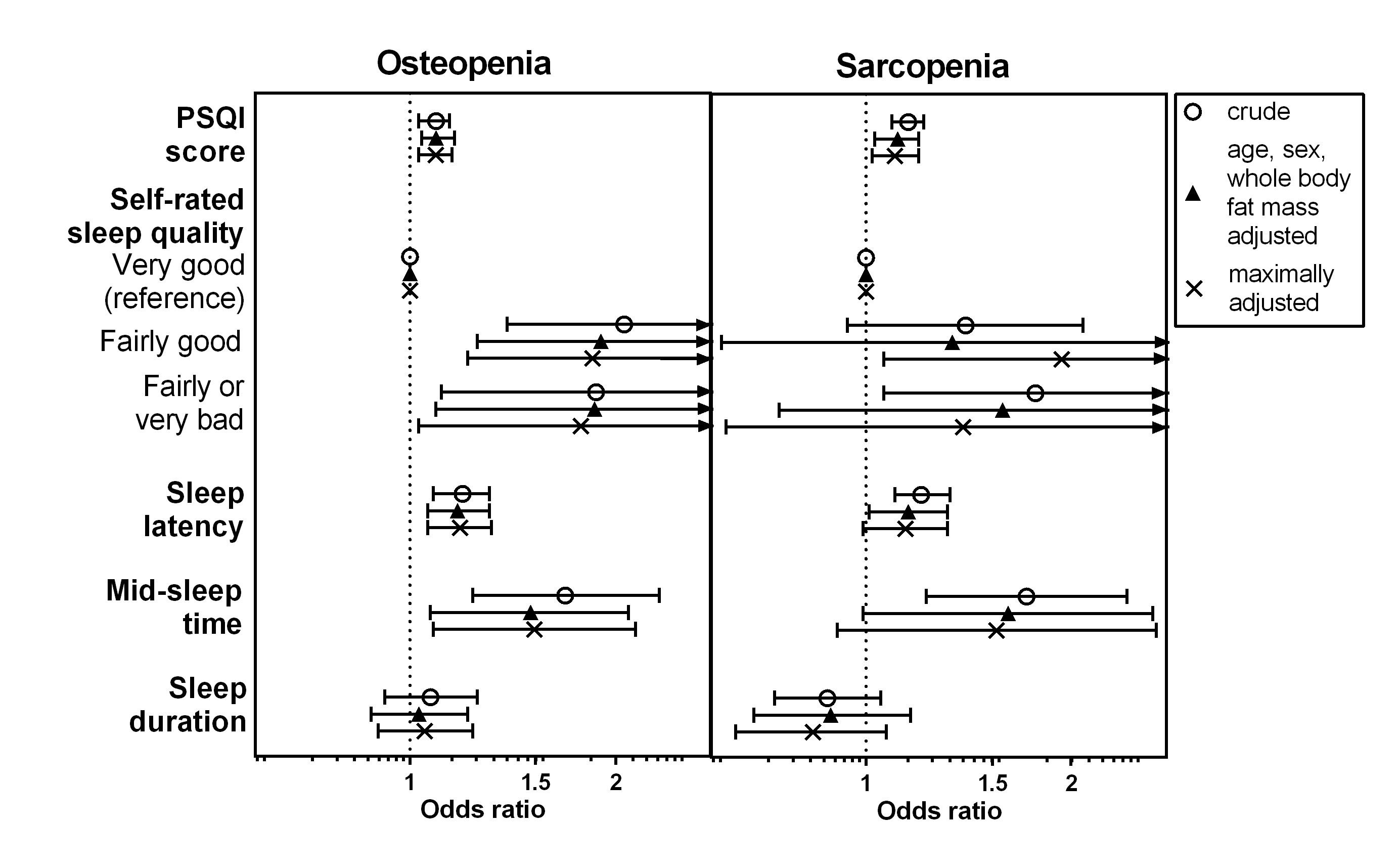
 Fig B:

OR (95% CI) for osteopenia and sarcopenia per unit change of sleep parameter. Maximally adjusted models are shown for reference (age, sex, whole body fat mass, ethnicity, education, alcohol intake, physical activity, vitamin D levels and season, usage of systemic corticosteroids and bisphosphonates (note that these models are also shown in **Fig. 1**)), and models were additionally adjusted for muscle mass, BMD spine, or BMD hip. PSQI, Pittsburgh Sleep Quality Index; BMD, bone mineral density. Results were based on analyses weighted towards the BMI distribution of the general population (n = 512 women, n = 403 men).
